# Characterization of acrosin and acrosin binding protein as novel CRISP2 interacting proteins in boar spermatozoa

**DOI:** 10.1111/andr.13413

**Published:** 2023-03-09

**Authors:** Min Zhang, Riccardo Zenezini Chiozzi, Elizabeth G Bromfield, Albert JR Heck, J Bernd Helms, Bart M Gadella

**Affiliations:** ^1^ Department of Biomolecular Health Sciences Faculty of Veterinary Medicine, Utrecht University Utrecht The Netherlands; ^2^ Biomolecular Mass Spectrometry and Proteomics Bijvoet Centre for Biomolecular Research and Utrecht Institute for Pharmaceutical Sciences Utrecht University Utrecht The Netherlands; ^3^ Netherlands Proteomics Centre Utrecht The Netherlands; ^4^ Priority Research Centre for Reproductive Science School of Environmental and Life Sciences, Discipline of Biological Sciences, University of Newcastle Callaghan New South Wales Australia

**Keywords:** boar spermatozoa, capacitation, colocalization, CRISP2, interacting proteins, protein complexes

## Abstract

**Background:**

Previously, we reported that cysteine‐rich secretory protein 2 is involved in high molecular weight complexes in boar spermatozoa. These cysteine‐rich secretory protein 2protein complexes are formed at the last phase of sperm formation in the testis and play a role in sperm shaping and functioning.

**Objectives:**

This study aimed to identify cysteine‐rich secretory protein 2 interacting partners. These binding partner interactions were investigated under different conditions, namely, non‐capacitating conditions, after the induction of in vitro sperm capacitation and subsequently during an ionophore A23187‐induced acrosome reaction.

**Materials and Methods:**

The incubated pig sperm samples were subjected to protein extraction. Extracted proteins were subjected to blue native gel electrophoresis and native immunoblots. Immunoreactive gel bands were excised and subjected to liquid chromatography–mass spectrometry (LC‐MS) analysis for protein identification. Protein extracts were also subjected to CRISP2 immunoprecipitation and analyzed by LC‐MS for protein identification. The most prominent cystein‐rich secretory protein 2 interacting proteins that appeared in both independent LC‐MS analyses were studied with a functional in situ proximity interaction assay to validate their property to interact with cystein‐rich secretory protein 2 in pig sperm.

**Results:**

Blue native gel electrophoresis and native immunoblots revealed that cystein‐rich secretory protein 2 was present within a ∼150 kDa protein complex under all three conditions. Interrogation of cystein‐rich secretory‐protein 2‐immunoreactive bands from blue native gels as well as cystein‐rich secretory protein 2 immunoprecipitated products using mass spectrometry consistently revealed that, beyond cystein‐rich secretory protein 2, acrosin and acrosin binding protein were among the most abundant interacting proteins and did interact under all three conditions. Co‐immunoprecipitation and immunoblotting indicated that cystein‐rich secretory protein 2 interacted with pro‐acrosin (∼53 kDa) and Aacrosin binding protein under all three conditions and additionally to acrosin (∼35 kDa) after capacitation and the acrosome reaction. The colocalization of these interacting proteins with cystein‐rich secretory protein 2 was assessed via in situ proximity ligation assays. The colocalization signal of cystein‐rich secretory protein 2 and acrosin in the acrosome seemed dispersed after capacitation but was consistently present in the sperm tail under all conditions. The fluorescent foci of cystein‐rich secretory protein 2 and acrsin binding protein colocalization appeared to be redistributed within the sperm head from the anterior acrosome to the post‐acrosomal sheath region upon capacitation.

**Discussion and Conclusion:**

These results suggest that CRISP2 may act as a scaffold for protein complex formation and dissociation to ensure the correct positioning of proteins required for the acrosome reaction and zona pellucida penetration.

## 1 INTRODUCTION

To fertilize an egg, spermatozoa must complete their final maturation by residing in the female reproductive tract to undergo a series of physiological and biochemical transformations termed capacitation.^1^, ^2^ Capacitation prepares the sperm to undergo an exocytotic process known as the acrosome reaction and perform vigorous flagellar movements characterized as hyperactivity.^3^ At the molecular level, capacitation involves several signaling pathways such as protein kinase A activation and protein tyrosine phosphorylation as well as lipid and protein reorganization.^4^, ^5^ Notably, sperm head surface remodeling, a capacitation‐associated event, has gained attention over the past decades. Specifically, the apical tip of the sperm head plasma membrane has been considered the primary site where capacitated (CAP) sperm bind to the oocyte zona pellucida (ZP).^6^ Capacitation leads to sperm‐ZP recognition by exposing ZP‐receptors on the sperm head surface that subsequently can bind to specific carbohydrates of the ZP proteins.^7^ In line with this, the transportation of acrosomal proteins, such as acrosin and acrosin binding protein (ACRBP), to the sperm head surface has been demonstrated in capacitating boar sperm.^8^ These proteins form high molecular weight complexes with a higher affinity for ZP binding.^8^ However, the mechanisms supporting acrosomal protein transportation during capacitation are still unclear. Besides this, it has also been reported that capacitation induces a concentration of lipid rafts at the apical tip of the sperm head surface.^9^ Sperm lipids rafts are liquid‐ordered, low‐density microdomains, serving as platforms for cell adhesion and signaling molecules.^8^, ^9^, ^10^ In capacitating boar sperm, the outer acrosomal membrane has been shown to dock with the sperm head plasma membrane by forming trimeric SNARE protein complexes.^11^, ^12^ This closer apposition of the two membranes is required for hybrid vesicle formation occurring during the acrosome reaction.^13^ Acrosomal exocytosis is a continuous process involving intermediate steps including progressive disassembly of the acrosomal matrix that is partially driven by proteolysis, for instance, acrosin cleavage.^14^, ^15^ Recently, it has been demonstrated that para‐crystalline patches of the acrosomal matrix start to fragment during capacitation.^16^ Intriguingly, there is evidence that some of the acrosome matrix proteins do interact with acrosomal membrane proteins that are reported to be involved in regulating acrosomal exocytosis.^17^, ^18^ Cysteine‐rich secretory protein 2 (CRISP2) is a member of the CRISP family that together with antigen 5 and pathogenesis‐related 1 proteins make up the CAP superfamily.^19^ Mammalian CRISP2 is exclusively expressed in the male reproductive tract and ultimately becomes an integral component of the sperm head and tail, the acrosome, the post‐acrosomal region, the fibrous tail structures, and the connecting piece.^20^, ^21^, ^22^, ^23^ CRISP2 possesses an evolutionary‐conserved CAP domain and a characteristic CRISP domain comprising a linker region and an ion channel regulatory (ICR) region. In vitro studies have shown that mouse CRISP2 can regulate Ca^2+^ flux via ryanodine receptor channels.^24^ CRISP2 has also been shown to bind with the cation channel of sperm 1 (CatSper1) subunit of the CatSper ion channel.^25^ Consequently, CRISP2 has been implicated in Ca^2+^‐related events such as sperm motility, capacitation, and the acrosome reaction.^26^, ^27^ Supporting this possibility, Crisp2^−/−^ sperm exhibit altered flagellum waveforms and an impaired ability to undergo a progesterone‐evoked acrosome reaction.^25^ In terms of mechanism, a yeast two‐hybrid screen of the adult mouse testis has been intensively used to identify mouse CRISP2 binding partners. CRISP2 is known to bind to the mitogen‐activated protein kinase kinase kinase 11 and gametogenetin 1 through its ICR domain.^28^, ^29^ Additionally, CRISP2 also binds to sperm head and tail‐associated protein (SHTAP), and this binding is attributed to the CAP domain.^30^ The CRISP2‐SHTAP complex is redistributed within the sperm head upon capacitation. Given that CRISP2 colocalizes with binding partners in the sperm head and sperm tail, a potential role for CRISP2 interacting complexes in the attainment of sperm function has been suggested. Nonetheless, those data are mostly derived from the mouse as an animal model. We previously found that boar sperm CRISP2 is present in high molecular weight complexes under a native state in both the perinuclear theca as well as in the outer dense fibers (ODF) and the fibrous sheath.^23^ Therefore, we now sought to identify CRISP2 interacting partners by affinity proteomics in boar sperm and follow their colocalization in situ prior to and during in vitro capacitation and the agonist‐induced acrosome reaction. We analyzed CRISP2‐protein complexes detected under native conditions and CRISP2‐targeted antigens by a quantitative, label‐free liquid chromatography‐mass spectrometry (LC‐MS/MS) approach.

## 2 MATERIALS AND METHODS

### 2.1 Reagents and antibodies

All chemicals were obtained from Sigma unless otherwise stated. Primary and secondary antibodies used in the present study are listed in Table S1.

### 2.2 Boar spermatozoa preparation

Semen was obtained from highly fertile boars from a commercial breeder (Cooperative Center for Artificial Insemination in Pigs, Veghel, the Netherlands). Semen was diluted to 20 million sperm/mL in a commercial extender, shipped in 80 mL sealed insemination tubes in a cool box (17°C) and used within 12 h of delivery. Diluted semen from three boars was pooled and washed through discontinuous Percoll (Cat#: 17089101, GE Healthcare) gradients as previously described.^23^

#### 2.2.1 In vitro capacitation and calcium ionophore A23187‐induced acrosomal exocytosis

The incubation media used in this study was a modified Tyrode's medium supplemented with bovine serum albumin (BSA), lactate and pyruvate.^31^ Capacitating medium consisted of 90 mM NaCl, 10.0 mM 4‐(2‐hydroxyethyl)‐1‐piperazine‐ethane‐sulfonic acid (HEPES), 3.0 mM KCl, 0.4 mM MgCl_2_, 2.0 mM CaCl_2_·2H_2_O, 0.3 mM Na_2_HPO_4_, 25 mM NaHCO_3_, 2.0 mM Na‐pyruvate, 5.0 mM D‐glucose, 21.6 mM Na lactate, 3.0 mg/mL fatty acid‐free BSA, 300 ± 10 mOsm/kg, pH 7.4. Medium supplied with neither CaCl_2_, NaHCO_3_ nor BSA was defined as non‐capacitating medium and an equimolar amount of NaCl was added to non‐capacitating medium to compensate for NaHCO_3_. Sodium pyruvate and BSA were added into the medium on the same day before use. Complete capacitating medium and non‐capacitating medium were equilibrated in the incubator (38.5°C, 5% CO_2_) with loose lids or in the water bath (38.5°C) with tight vials for at least 2 h. Percoll‐washed sperm were resuspended in capacitating medium (1 mL, 20 × 10^6^ sperm/mL) in open tubes and incubated for 2.5 h in the incubator or incubated in non‐capacitating medium (1 mL, 20 × 10^6^ sperm/mL) in air‐tight tubes for 2.5 h at 38.5°C in a pre‐warmed water bath. During the last 30 min of capacitation, sperm cells were exposed to 5 µM calcium ionophore A23187 to induce acrosome exocytosis and incubated under the same conditions as capacitation. All sperm incubations were carried out in 5 mL polystyrene round bottom tubes (Falcon, 352054; Life Sciences). At least five tubes were incubated for each condition. After incubation, sperm cells were spun down and washed twice with phosphate‐buffered saline (phosphate buffered salt [PBS]; 137 mM NaCl, 8.0 mM Na_2_HPO_4_·2H_2_O, 1.5 mM KH_2_PO_4_, 2.7 mM KCl, pH 7.4) at 750 x g for 10 min, room temperature (RT). Sperm cells were either fixed for immunofluorescence or stored at −80°C.

### 2.3 Blue native polyacrylamide gel electrophoresis (PAGE) and native blots

Non‐capacitated (NC), CAP, and ionophore‐induced (II) sperm cells were lysed in a commercial lysis buffer (Cat#: 88805, Thermo Scientific) with freshly added protease inhibitors: aprotinin, leupeptin, pepstatin, and phenylmethanesulfonyl fluoride (phenyl methane sulfonyl fluoride [PMSF]; Thermo Scientific) for 30 min on ice with mixing, followed by a 15 min centrifugation at 14,000 x g at 4°C. Supernatants were recovered and mixed with 4x native sample buffer (400 mM Tris HCl, pH 8.6, 40% glycerol, 0.04% Brom‐phenol‐Blue) and 5% G‐250 sample additive (Cat#: BN2004, Thermo Scientific) before loading onto 4%∼20% precast gels (Cat#: 4561094, Bio‐Rad). Native running buffer (25 mM Tris‐base, 192 mM glycine, pH 8.3) and native PAGE cathode buffer additive (20x; Cat#: BN2002, Thermo Scientific) were used to make cathode running buffer following the manufacturer's instructions. Electrophoresis was carried out at 150 V, RT for 2∼2.5 h. Gels were either stained with Coomassie R‐250 or prepared for native blots.

Native immunoblotting was carried out as in conventional western blotting procedures with the exception that the transfer buffer was a tris‐glycine buffer (12 mM tris‐base, 96 mM glycine, pH 8.3). Proteins were blotted onto 0.45 µm polyvinylidene difluoride membranes (GE Healthcare) at 25 V, RT for 1 h. After transfer, membranes were fixed in 8% (v/v) acetic acid for 15 min and rinsed with water, then air‐dried overnight at RT. Dried membranes were rewet in pure methanol to remove excessive bound dye. After rinsing with water, membranes were continued from the blocking step as conventional western blotting immunodetection (see immunoblotting). NativeMark unstained protein standard (LC0725, Thermo Scientific) was used to estimate protein size.

### 2.4 Co‐immunoprecipitation (Co‐IP)

To identify potential CRISP2 interacting proteins, we employed a reciprocal Co‐IP assay using a Crosslink Magnetic IP/Co‐IP kit (Cat#: 88805, Thermo Scientific). CRISP2 antibody (10 µg per reaction) was cross‐linked to protein A/G magnetic beads according to the manufacturer's instructions. Sperm lysates were prepared as described above for blue native PAGE. Crosslinked beads and approximately 0.5 mg of sperm lysates were incubated at 4°C overnight with agitation. The beads were collected and washed three times before the elution of bound proteins. The supernatants containing targeted antigens were saved for immunoblotting or MS analysis.

### 2.5 Protein identification by LC‐MS from blue native PAGE and CRISP2‐precipitated antigens

CRISP2 interacting protein complexes were carefully excised from gels (Figure S2A) and CRISP2‐targeted antigens from the co‐IP eluate were prepared for LC‐MS analysis. The efficiency of CRISP2 pull‐down assays was validated by immunoblotting (Figure S2B). The gel slices were first de‐stained then reduced with dithiothreitol and alkylated with iodoacetamide. All gel bands were then digested overnight with trypsin. Peptides from all samples were analyzed using an Ultimate3000 high‐performance liquid chromatography system (Thermo Scientific) coupled online to a Q Exactive HF‐x mass spectrometer (Thermo Scientific) as previously described.^32^ Buffer A consisted of water acidified with 0.1% formic acid, while buffer B was 80% acetonitrile and 20% water with 0.1% formic acid. The peptides were first trapped for 1 min at 30 µL/min with 100% buffer A on a trap (0.3 mm by 5 mm with PepMap C18, 5 µm, 100 Å; Thermo Scientific); after trapping, the peptides were separated by a 50 cm analytical column packed with C18 beads (Poroshell 120 EC‐C18, 2.7 µm; Agilent Technologies). The gradient was 9% to 40% B in 40 min at 400 nL/min. Buffer B was then raised to 55% in 2 min and increased to 99% for the cleaning step. Peptides were ionized using a spray voltage of 1.9 kV and a capillary heated at 275°C. The mass spectrometer was set to acquire full‐scan MS spectra (350 to 1400 m/z ratio) for a maximum injection time of 120 ms at a mass resolution of 60,000 and an automated gain control (AGC) target value of 3 × 10^6^. Up to six of the most intense precursor ions were selected for MS/MS. Higher energy collission dissociation (HCD) fragmentation was performed in the HCD cell, with the readout in the Orbitrap mass analyzer at a resolution of 30,000 (isolation window of 1.4 Th) and an AGC target value of 1 × 10^5^ with a maximum injection time of 54 ms and a normalized collision energy of 27%. The raw files were analyzed with MaxQuant (version 2.0.3) with all the default settings adding deamidation (NQ) and phosphorylation (STY) as dynamic modifications against the Sus scrofa sperm‐specific proteome (from proteins identified in Zhang et al., 2022^33^) containing 2474 entries adding common contaminants. MaxQuant was used with the standard parameters adding only the “iBAQ Quantification” and “Match between runs” with automatic values. High‐confidence positive identifications were based on a minimum of two matching peptides.

### 2.6 SDS‐PAGE and immunoblotting

Percoll‐washed sperm cells were sonicated and sperm heads and tails were isolated as described previously.^23^ Whole sperm cells, sperm heads and tails were lysed in radio immune preciptation assay buffer (Thermo Scientific) with freshly added protease inhibitors: aprotinin, leupeptin, pepstatin, and PMSF (Thermo Scientific) on ice for 30 min followed by a 15 min spin at 14,000 x g, 4°C. Sperm lysates or CRISP2 precipitates were denatured in 4x sodium dodecyl sulfate (SDS) sample buffer (200 mM Tris‐HCl, pH 6.8, 10% β‐mercapto‐ethanol, 8% SDS, 0.08% bromophenol blue, 40% glycerol) and boiled for 10 min. Samples were loaded onto SDS‐PAGE gels (5% stacking gel, 12% resolving gel) and were blotted onto 0.2 µm nitrocellulose membranes (GE Healthcare) at 100 V for 1 h. After blocking for 3 h at RT in 5% (w/v) BSA in PBS with 0.05% (v/v) Tween‐20 (PBST), membranes were incubated with primary antibodies (Supplementary Table S1) overnight at 4°C. After three washes in PBST for 15 min, membranes were incubated with horse radish peroxidase conjugated secondary antibodies (Table S1) for 1 h at RT. After rinsing four times in PBST for 20 min, membranes were developed using chemiluminescence (enzyme chemilumenescence [ECL]‐detection kit; Supersignal West Pico, Pierce). Migration levels of proteins were visualized using a pre‐stained protein ladder, 10 to 250 kDa (Thermo Scientific).

### 2.7 Immunolocalization of CRISP2 interacting proteins in boar sperm

Protein colocalization was assessed via in situ proximity ligation assays (PLAs). For the colocalization of CRISP2 and acrosin, sperm cells were fixed and permeabilized in −20°C methanol for 5 min, diluted in PBS and deposited on slides. Sperm cells were blocked and incubated with primary antibodies, overnight at 4°C, followed by appropriate secondary antibodies conjugated with oligonucleotides (Duolink PLA Probe Anti‐Rabbit PLUS, Cat#: DUO92002, Anti‐Mouse MINUS, Cat#: DUO92004, Sigma). After enzymatic ligation and amplification, target proteins were colocalized with peanut agglutinin lectin (PNA) via dual labeling with Alexa Flour 488. Colocalization of CRISP2 and ACRBP was performed using Duolink PLA Probemaker kit (Cat#: DUO96020, Sigma) following the manufacturer's instructions. Briefly, sperm cells were fixed in 4% paraformaldehyde (PFA) and permeabilized in cold acetone for 10 min, washed and blocked before incubating with primary antibodies conjugated with oligonucleotides (PLA probes), overnight at 4°C. After enzymatic ligation and amplification, target proteins residing within a maximum distance of 40 nm were identified by the production of discrete fluorescent foci.^34^, ^35^, ^36^ Additionally, conventional single protein immunostaining was performed following standard protocols.^33^ In all cases, fluorescent labeling of sperm cells was visualized with a Leica SPE‐II confocal microscope using a 63x objective (NA 1.3, HCX PLANAPO oil). Scale bars were added using Image J software (bundled with 64‐bit Java 1.8.0_172, National Institutes of Health).

## 3 RESULTS

### 3.1 Identification of CRISP2 interacting proteins in boar spermatozoa

**FIGURE 1:**
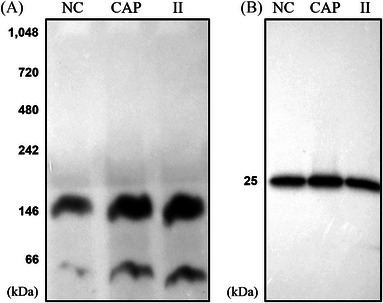
Cysteine‐rich secretory protein 2 (CRISP2) is involved in a ∼150 kDa protein complex under native conditions. After incubation and washing, non‐capacitated (NC), capacitated (CAP), and ionophore‐induced (II) sperm cells were lysed and prepared for blue native polyacrylamide gel electrophoresis (PAGE) and SDS‐PAGE. (A) Native blots on the lysates from NC, CAP, and II sperm cells probed with anti‐CRISP2 antibody. (B) Lysates from NC, CAP, and II sperm cells were mixed with 4X SDS sample buffer, cooked and analyzed by western blots. Three ejaculates from different boar were mixed as one biological replicate and this experiment was replicated three times.

Native blots revealed that anti‐CRISP2 recognized a ∼150 kDa protein complex in boar sperm, irrespective of whether lysates were extracted from NC, capacitated (CAP), or II sperm cells (Figure 1A). Of note, there was an additional lower band (∼50 kDa) that likely corresponds to dimeric CRISP2 under non‐denaturing conditions (Figure 1A). Note that in a previous study (Zhang et al., 2021), a different sourced anti‐CRISP2 antibody was used, which was immunized against 13 amino acids of CRISP2, and it recognized an additional ∼200 kDa on native blots.^23^ It is possible that a unique conformational epitope under native state was recognized by this specific antibody. Western blots from SDS‐PAGE gels demonstrated that CRISP2 was present as a ∼25 kDa monomer under reducing conditions with no obvious difference in the amount of CRISP2 among the three samples (Figure 1B). Additionally, Coomassie brilliant blue staining of SDS‐PAGE gels was conducted to show the total protein loading (Figure S1A), and western blots probed with anti‐alpha tubulin were supplied in Figure S1C. NC, CAP, and II sperm cells were processed, and two methods were used to isolate CRISP2 interacting protein complexes. (i) Protein samples were run through a native gel, immunoblotted with anti‐CRISP2, and the 150 kDa band was excised from the gel (Figure S2A) and used in a proteomics workflow. (ii) CRISP2 interacting protein complexes were immunoprecipitated and the eluate was then used in the proteomics workflow.

As an initial validation, we examined the proteins from native PAGE gel plug samples of the 150 kDa band of NC, CAP, and II sperm samples after detection by MS (Supplementary Table S2). MS analysis of CRISP2‐pulldown eluates from NC, CAP, and II sperm samples revealed that CRISP2 was detected among the most abundant proteins in the eluate. In addition to CRISP2, we consistently detected the proteins acrosin, and ACRBP among the most abundant proteins within each sample group (Supplementary Table S2). Notably, in the CAP sample, acrosin, ACRBP and CRISP2 were detected as the top four most abundant proteins in the pulldown, with their collective molecular weight ∼150 kDa. Thus, we choose to further characterize these identifications (IDs) as putative constituents of the 150 kDa complex shown in Figure 1A. We reduced the list of IDs by comparing those obtained through the 150 kDa gel plugs with those obtained through CRISP2 IP. The common proteins that were consistently present through the use of both techniques were ranked by abundance. The list revealed the presence of CRISP2, acrosin, and ACRBP thus providing credence for their further analysis. The top 12 proteins of this common protein list are represented and categorized based on their function in Table 1.

**TABLE 1:** Top 12 most abundant cysteine‐rich secretory protein 2 (CRISP2)‐interacting partners identified by mass spectrometry in both excised CRISP2 native gel complexes and CRISP2 immunoprecipitation eluates.

**Protein IDs**	**Protein name/functional category**
A0A287AFN9	**Acrosomal proteins** Acrosin (ACR)
Q29016	Acrosin binding protein, 60 kDa form (ACRBP)
I7HJH6	**Spermadhesins** AQN‐3
Q4R0H8	AWN
P26461	**Protease‐related** Sperm‐associated acrosin inhibitor (AI)
A0A287BEN1	Serine protease inhibitor kazal‐type 2 isoform 1 (SPINK2)
A0A287A7G8	**Histones** Histone H2B
P62802	Histone H4
A0A287ATN8	**Chaperones and proteins with a mitochondrial function** 60 kDa chaperonin, 60 kDa heat shock protein, mitochondrial (HSPD1)
F1SMZ6	10 kDa chaperonin, 10 kDa heat shock protein, mitochondrial (HSPE1)
A0A287BPT0	Amine oxidase (IL4I1)
P63053	**Ubiquitin** Ubiquitin A‐52 residue ribosomal protein fusion product 1 (UBA52)

### 3.2 Boar sperm acrosin and ACRBP co‐immunoprecipitate with CRISP2

**FIGURE 2:**
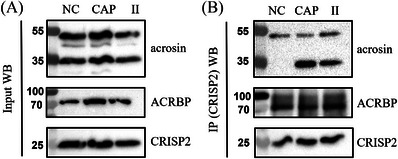
Validation of acrosin and acrosin binding protein (ACRBP) as CRISP2 interacting partners by western blots analysis of CRISP2 immunoprecipitation (IP). CRISP2 antibody was cross‐linked to protein A/G magnetic beads and incubated with lysates from NC, CAP, and II sperm cells overnight at 4°C. After intensive washing, CRISP2 and CRISP2‐associated proteins were disassociated from the magnetic beads. (A) Sperm lysates before incubation with the beads were saved and analyzed by western blots. (B) CRISP2 pull‐down products were analyzed by western blots probed with anti‐acrosin, anti‐ACRBP, and anti‐CRISP2 antibodies. Full western blots images are shown in Supplementary Figure S3.

Next, the putative interactions between CRISP2 and acrosin/ACRBP were validated using biochemical techniques. First, Co‐IP using the CRISP2 antibody resulted in the detection of acrosin and ACRBP in the lysates of NC, CAP, and II sperm cells (Figure 2A). Immunoblotting analysis of CRISP2 co‐immunoprecipitates (eluates) probed with the CRISP2 antibody repeatedly showed that CRISP2 was present in the pull‐down products indicating an efficient isolation (Figures 2B and S2B). Probing with the acrosin antibody revealed that proacrosin (∼53 kDa) was consistently present in the three conditions, while acrosin (∼35 kDa) interaction with CRISP2 was only observed after CAP and II (Figure 2B). The ACRBP antibody used in our study was against the N‐terminal part (aa 205–220) of the human ACRBP allowing it to recognize the ACRBP precursor or the fragments containing the N‐terminal part but not the mature form (viz., sp32) without the N‐terminal half.^37^ Application of this antibody confirmed its presence in all three samples as well as in the CRISP2 pull‐down products (Figure 2A,B).

### 3.3 Subcellular localization of acrosin and ACRBP in boar spermatozoa

**FIGURE 3:**
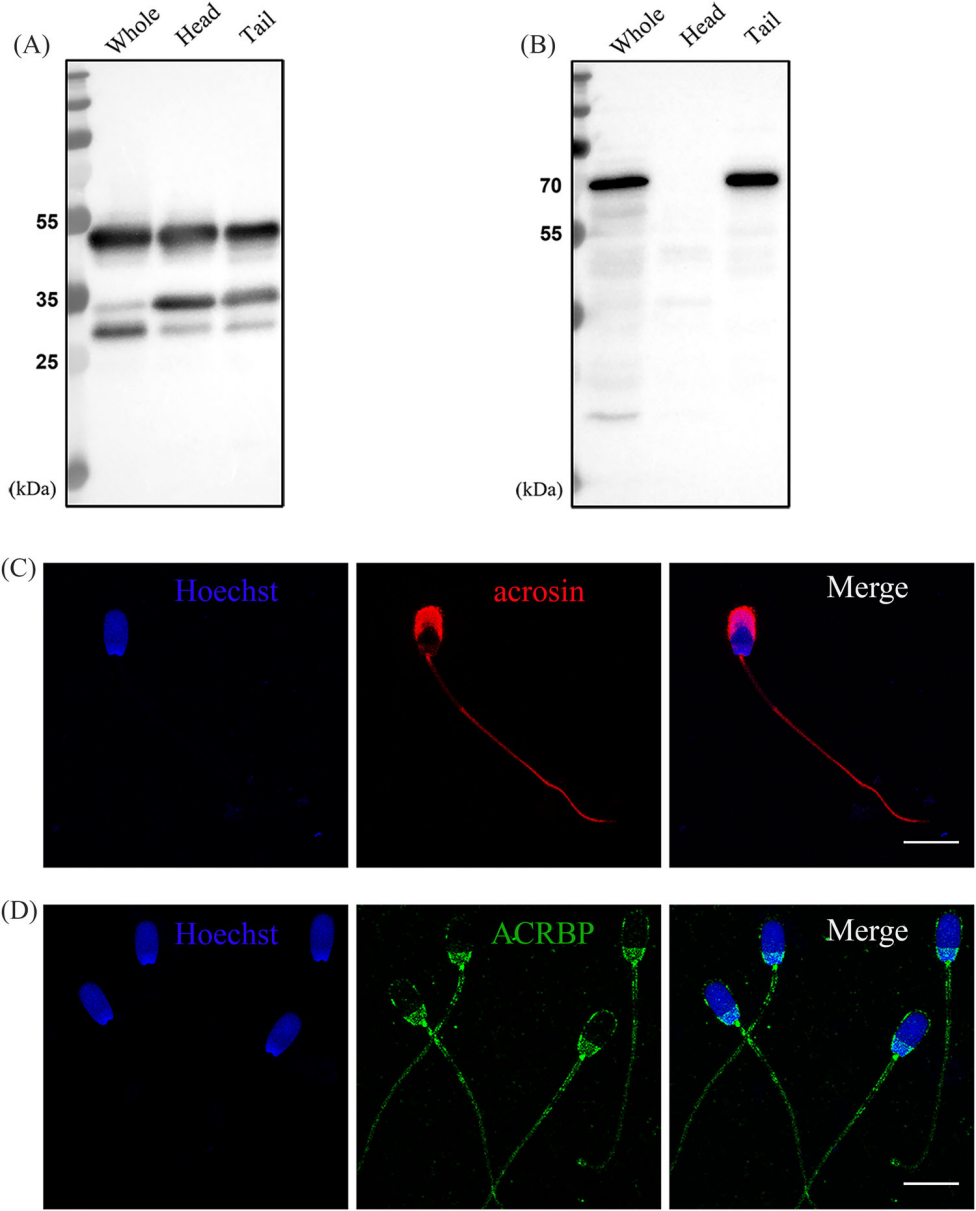
Subcellular localization of acrosin and ACRBP in boar spermatozoa. Western blots of acrosin (A) and ACRBP (B) on the lysates from whole sperm, sperm heads, and tails. (C) Percoll‐washed sperm cells were fixed in −20°C methanol for 5 min, incubated with anti‐acrosin followed by Alexa Flour 568 conjugated secondary antibody (red), counterstained with Hoechst 33342 (blue). (D) Sperm cells were fixed in 4% paraformaldehyde (PFA) and permeabilized in cold acetone for 10 min, incubated with anti‐ACRBP followed by Alexa Flour 488 conjugated secondary antibody (green) and counterstained with Hoechst 33342 (blue). This experiment was replicated three times. Scale bar = 10 µm.

To gain a better understanding of the biochemical features of acrosin and ACRBP in boar spermatozoa, western blots of the lysates from ejaculated whole sperm, purified sperm heads and sperm tails were isolated and analyzed. In parallel to the western blots of Figure 3 complementary SDS‐PAGE gels were stained with Coomassie brilliant blue to show the total protein loading for whole sperm and the head and tail subfractions (Figure S1B) and western blots probed with anti‐alpha tubulin was supplied in Figure S1D. The results showed that the ∼53 kDa proacrosin was the predominant form present in the ejaculated boar spermatozoa (Figure 3A). Two bands corresponding to ∼35 kDa and ∼28 kDa acrosin were also observed in whole sperm. Immunoblots on the extracts from purified sperm head and tail populations revealed that the ∼53 kDa proacrosin and ∼35 kDa acrosin were the two major forms associated with sperm heads and tails (Figure 3A). Western blot analysis of ACRBP on the same samples showed that ACRBP was undetectable in the lysates from sperm heads; however, it was abundantly associated with the sperm tail fractions (Figure 3B).

The localization of acrosin and ACRBP in boar spermatozoa was further investigated by immunofluorescence microscopy. It showed that acrosin immunofluorescence was localized in the sperm acrosome (Figure 3C). Additionally, faint fluorescence was observed in the connecting piece and the principal piece of the sperm tail. Immunolocalization of ACRBP showed that ACRBP fluorescence was present in the plasma membrane and the post‐acrosomal region of the sperm head as well as the entire sperm tail (Figure 3D).

### 3.4 CRISP2 colocalizes with acrosin and ACRBP in boar sperm

**FIGURE 4:**
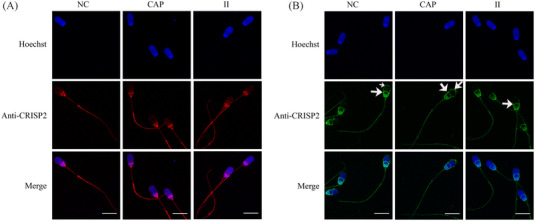
Immunolocalization of CRISP2. (A) NC, CAP, and II sperm cells were fixed in 4% PFA and permeabilized in cold acetone for 10 min, incubated with anti‐CRISP2 followed by Alexa Flour 568 conjugated secondary antibody (red) and counterstained with Hoechst 33342 (blue). (B) Immunofluorescent staining of CRISP2 on sperm cells fixed in −20°C methanol for 5 min, incubated with anti‐CRISP2 followed by Alexa Flour 488 conjugated secondary antibody (green) and counterstained with Hoechst 33342 (blue). Arrows indicated additional CRISP2 signals were observed. Three ejaculates from different boar were mixed as one biological replicate, and this experiment was replicated three times. Scale bar = 10 µm.

**FIGURE 5:**
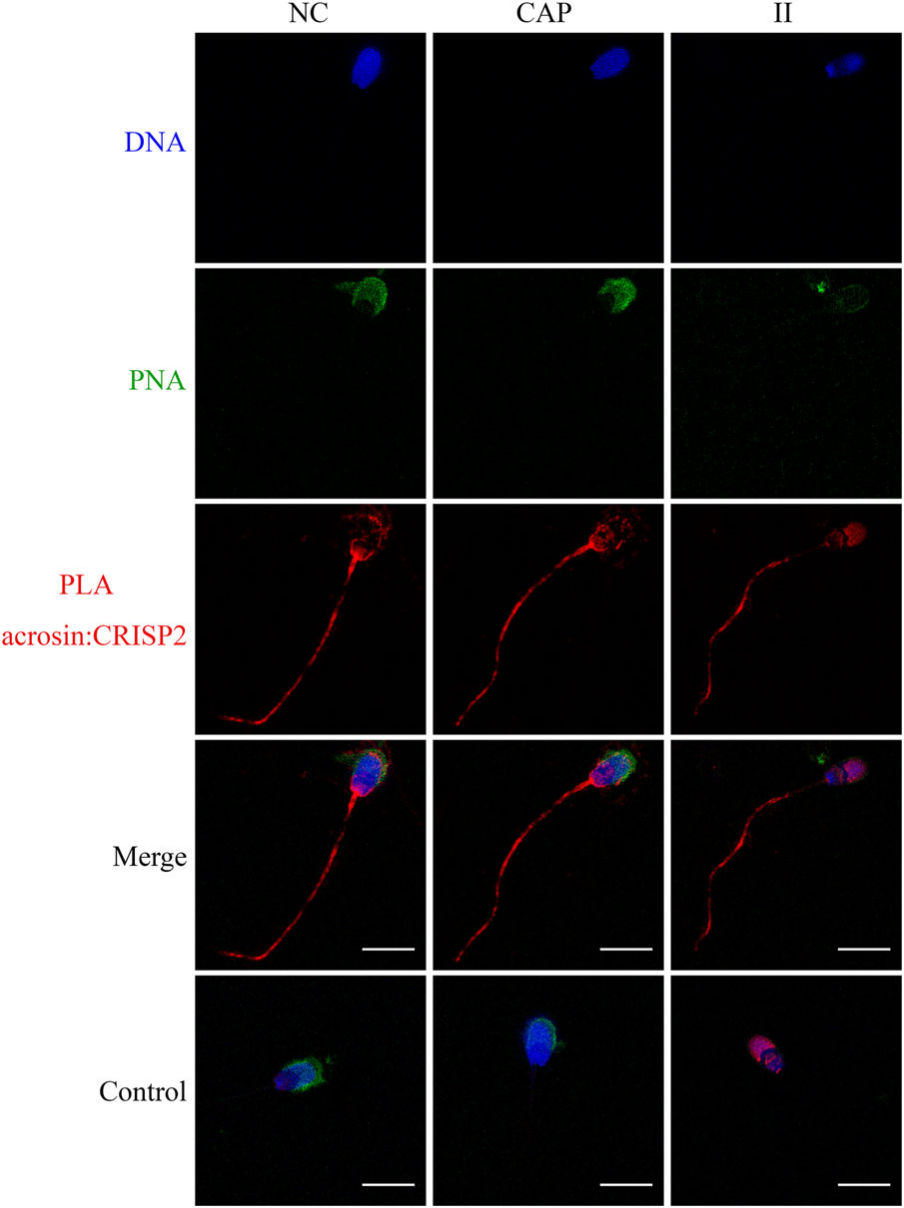
Colocalization of acrosin and CRISP2 via an in situ proximity ligation assay (PLA). NC, CAP, and II sperm cells were permeabilized in −20°C methanol for 5 min, incubated with anti‐acrosin and anti‐CRISP2 antibodies, followed by appropriate secondary antibodies conjugated with oligonucleotides. Sperm cells were counterstained with peanut agglutinin lectin (PNA; green) and Hoechst 33342 (blue). Primary antibodies were omitted in control. Three ejaculates from different boar were mixed as one biological replicate, and this experiment was replicated twice. Scale bar = 10 µm.

**FIGURE 6:**
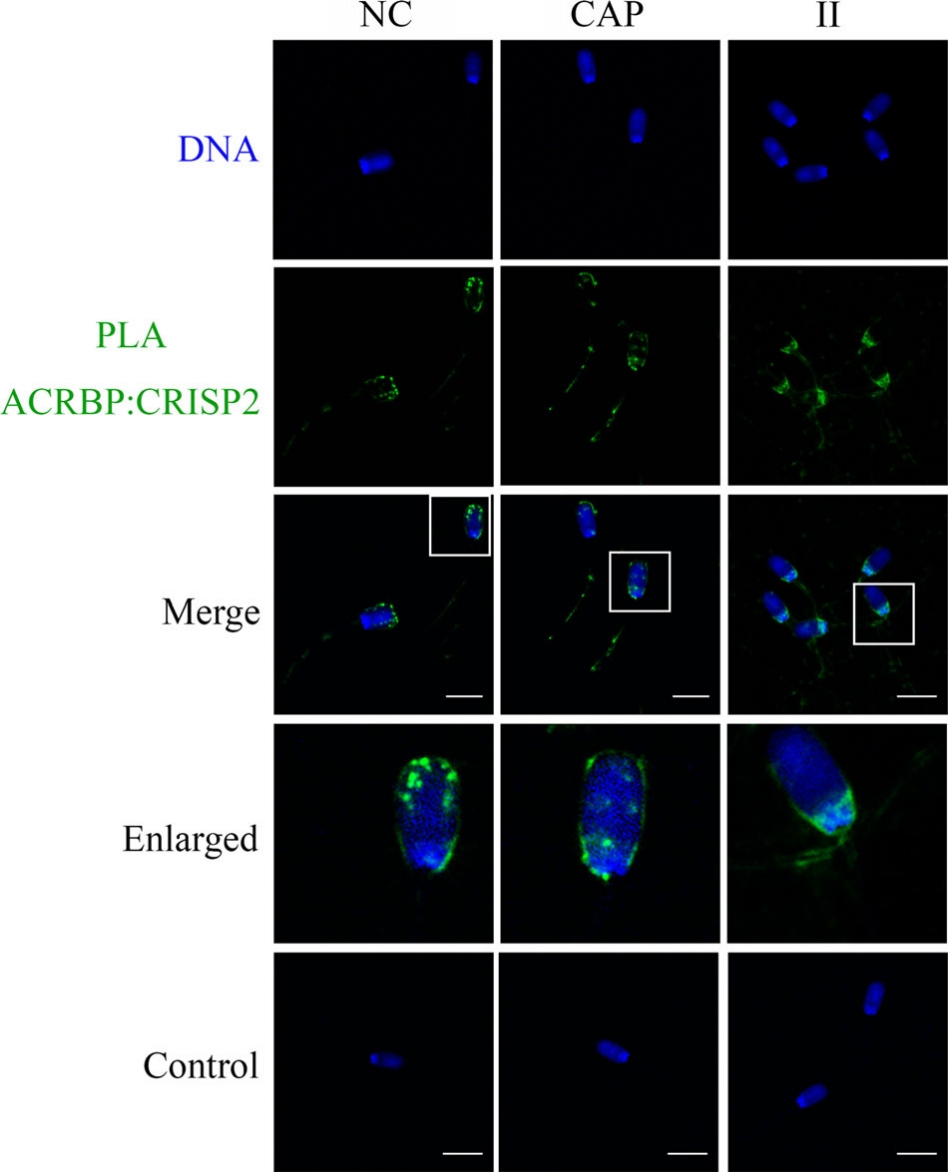
Colocalization of ACRBP and CRISP2 via PLA. Sperm cells were fixed in 4% PFA and permeabilized in cold acetone for 10 min, incubated with primary antibodies conjugated with oligonucleotides, and counterstained with Hoechst 33342 (blue). Three ejaculates from different boar were mixed as one biological replicate, and this experiment was replicated twice. Primary antibodies were omitted in control. Scale bar = 10 µm.

The conventional immunofluorescence analysis of CRISP2 was performed after 4% PFA fixation, followed by cold acetone permeabilization or after a fixation/permeabilization in methanol. The former method revealed a consistent staining pattern of CRISP2 in the post‐acrosomal region, the connecting piece, and the sperm tail in NC, CAP, and II populations (Figure 4A). However, methanol preparation of sperm resulted in additional labeling of the equatorial segment in all the sperm cells and faint fluorescence was also observed on the apical ridge of the sperm head in NC and CAP sperm cells (Figure 4B). In situ colocalization of CRISP2 and acrosin was investigated by an indirect PLA in which the PLA probes were connected to the secondary antibodies. Fluorescent PNA was used to assess the acrosome integrity. CRISP2‐acrosin interactions were reflected by red PLA signal and were found associated with the post‐acrosomal region and the inner acrosome membrane in NC sperm and as discrete foci of colocalization across the acrosome of CAP sperm (Figure 5). PLA foci were also consistently present in the sperm tail (Figure 5). Colocalization of CRISP2 and ACRBP was performed via a direct PLA assay in which the PLA probes were linked to the primary antibodies.^36^ Intense green fluorescent foci were observed over the anterior acrosome (AA) in NC sperm, and these discrete foci of colocalization were translocated to the post‐acrosomal sheath (PAS) region after CAP and II incubation (Figure 6). The pattern of CRISP2 and ACRBP foci redistribution in NC, CAP, and II sperm cells was quantified (Table 2). Our data revealed that 87.7% of NC sperm have the green foci in the AA, whereas 12.3% of the NC sperm have a labeling pattern in the PAS region. The percentage of sperm that have the foci redistribution to the PAS region was increased to 81.4% after capacitation and 85.5% after the acrosome reaction.

**TABLE 2:** Analysis of CRISP2‐ACRBP foci redistribution within the sperm head from the anterior acrosome (AA) to the post‐acrosomal sheath (PAS) region in non‐capacitated (NC), capacitated (CAP), and ionophore‐induced (II) sperm groups

**Group**	**No. (%) spermatozoa show an AA staining pattern**	**No. (%) spermatozoa show a PAS staining pattern**	**No. spermatozoa examined**
NC	265(87.7%)	37(12.3%)	302
CAP	60(18.6%)	263(81.4%)	323
II	51(14.5%)	300(85.5%)	351

*Note*: Semen from three different boars were pooled and washed for sperm in vitro incubation. This experiment was replicated twice. At least 150 sperm cells were counted for each group from one independent experiment.

## 4 DISCUSSION

In this article, we demonstrated that boar sperm CRISP2 was consistently present in a ∼150 kDa protein complex under native conditions, irrespective of the sperm functional status (NC, CAP, or II). The ∼50 kDa band on native blots was likely a dimeric form of CRISP2. This property is a feature of other CAP proteins, for instance, Golgi‐associated plant pathogenesis‐related protein 1 is present as a dimer in solution.^38^ Therefore, our data suggest that CRISP2 may function as a dimer within sperm. The dimerization of CRISP2 is likely to be the result of the conserved CAP domain.^39^ We were interested in the isolated CRISP2 complex and sought to identify CRISP2 interacting proteins in boar sperm and investigate the colocalization of target proteins following capacitation and acrosome reaction. Proteomic analysis of the CRISP2 complex under native conditions as well as the eluate of a CRISP2 pull‐down showed that besides CRISP2, acrosin and ACRBP were detected as abundant proteins by both techniques and were highly abundant (Supplementary Table S2). Some caution has to be taken in consideration when working on excised native protein bands (in this case, the molecular weight of 150 kDa band that was on the western blot was shown to contain CRISP2). (i) Other proteins (complexes) may contaminate this excised gel area, and this could contaminate the identified proteins as listed in Supplementary Table S2, as they may not interact with CRISP2. (ii) Furthermore, during preparations of the different sperm fractions rearrangements of protein–protein interactions may take place, which could result in the formation of other CRISP2‐positive bands. For instance, in a previous study of us,^23^ sonicated sperm tail and head samples showed a weak positive CRISP2 immune staining of a 200 kDa band, while this 200 kDa CRISP2 containing protein complex was not observed in whole (not sonicated) sperm samples. We, therefore, choose to excise only the area just around the consistent 150 kDa band (the samples of the present study were not sonicated and did not contain the previously reported 200 kDa CRISP‐positive protein complex). Due to these limitations in native protein gel electrophoresis, we also identified CRISP2 interacting proteins with a CRISP2 pull‐down assay. The two techniques consistently gave the same identification of the most abundant proteins.

### 4.1 Characterization of acrosin and ACRBP in boar spermatozoa

Acrosin acts as a zona lysin during zona penetration and is not strictly necessary on its own for sperm zona penetration, given that acrosin‐deficient mice and rats are fertile.^40^, ^41^ While, sperm from acrosin‐deficient models exhibited delayed fertilization.^41^, ^42^ However, in other species such as the golden hamster, acrosin‐deficient sperm cannot achieve sperm‐zona penetration.^43^ Therefore, the exact role of acrosin in sperm‐zona penetration events is not yet clear. Other undefined proteases or proteins may compensate for the acrosin^−/−^ phenotype during sperm‐zona interaction. Acrosin is localized inactively in the sperm acrosome as a soluble constituent as well as in a particulate fraction forming the acrosomal matrix.^16^, ^44^ Evidence indicates that acrosin is also associated with acrosomal membranes.^44^, ^45^ Biochemically, acrosin is detected as proacrosin (∼53–55 kDa), intermediate forms (∼45–49 kDa), mature acrosin (∼35 kDa), and other forms.^46^, ^47^ In our study, we demonstrated that the ∼53 kDa proacrosin form was the predominant form in the extracts of boar spermatozoa. Interestingly, we also observed that a sizeable portion of proacrosin and mature acrosin was associated with sperm head fractions. A similar phenomenon has been reported for bull sperm acrosin.^45^ Surprisingly, proacrosin and acrosin were also found with the sperm tail fractions, and it appeared that the ∼53 kDa proacrosin was the major form. Immunofluorescent signals of anti‐acrosin in the sperm tail supported our immunoblotting results. In fact, previous proteomic analysis of human and pig sperm tails also demonstrates that acrosin contributes to the sperm tail proteome.^33^, ^48^ The detection of acrosin immunofluorescence in the sperm tail was made possible by a rather harsh permeabilization method, which led to the exposure of the antigen epitope. Immunoblots of extracts from CAP and II sperm showed no obvious conversion of ∼53 kDa proacrosin into ∼35 kDa acrosin following CAP and II treatments. This may be explained by the autocatalytic mechanism of proacrosin, which can undergo auto‐cleavage into other forms of acrosin after solubilization from the sperm cells.^49^, ^50^ Additionally, and in line with our results, a previous study in rabbit sperm revealed that the bulk of proacrosin remained unprocessed during capacitation and the calcium ionophore A23187‐induced acrosome reaction.^51^

Porcine ACRBP is synthesized as a precursor and processed into a mature form (sp32) by removal of the N‐terminal half of the ACRBP.^52^ sp32 has been intensively investigated because it is a major protein that is phosphorylated during capacitation^53^ and involved in sperm‐zona interaction.^54^ Moreover, sp32 phosphorylation levels correlate with the conversion of proacrosin into mature acrosin.^55^ However, the fate of sperm ACRBP after ejaculation is barely studied. The present study has focused on the biochemical features of boar sperm ACRBP after ejaculation using an antibody against the central part of the N‐terminal sequence. Our results demonstrate that the precursor ACRBP was still present in ejaculated boar spermatozoa and associated with the sperm tail fractions after sonication. Immunostaining of ACRBP indicated the localization of ACRBP in the plasma membrane as well as the post‐acrosomal region of the sperm head. This indicates that the absence of ACRBP on the immunoblots of sperm head fractions was likely caused by sonication. Consistent with our study, Zigo et al. report the localization of boar ACRBP in the post‐acrosomal region and the midpiece of the sperm.^56^ Specifically, in addition to the wild‐type Acrbp mRNA expressed in pig, human, and guinea pig, there exists another intron 5‐retaining variant Acrbp mRNA termed ACRBP‐V5 in mice.^37^ ACRBP‐V5 is essential for packaging proacrosin into acrosomal granules for normal acrosome formation during early spermiogenesis, and ACRBP retains proacrosin inactively in the acrosome until acrosomal exocytosis.^57^ It is still unclear how ACRBP is processed into sp32 due to the fact that ACRBP itself does not have a strong consensus site for convertase cleavage, and ACRBP does not undergo proteolytic processing in proprotein convertase 4 null mice.^58^ ACRBP processing into sp32 may be regulated by other yet unknown proteases. Thus, we propose that a population of ACRBP is likely not processed into sp32 during epididymal maturation. Instead, this ACRBP population is probably stabilized by interacting with other proteins or by other post‐translational modifications, for example, phosphorylation, as occurred to the sp32 during capacitation.^53^

### 4.2 Colocalization of CRISP2 target proteins in boar sperm

IP blots showed that the ∼53 kDa proacrosin was consistently present in CRISP2 pull‐down products from the lysates of NC, CAP, and II sperm cells. Interestingly, the absence of ∼35 kDa acrosin in CRISP2 precipitates from the lysates of NC sperm cells, and emerging in CRISP2 precipitates from the lysates of CAP and II sperm cells indicates that the interaction between CRISP2 and the ∼35 kDa acrosin is an event associated with capacitation. The fact that ∼35 kDa acrosin was present in total lysates of NC sperm cells excludes the possibility that CRISP2 interacted with the ∼35 kDa acrosin produced by the autocatalytic reaction of the ∼53 kDa proacrosin. On the contrary, it suggests that there was indeed an efficient conversion of ∼53 kDa proacrosin into ∼35 kDa acrosin during capacitation and the acrosome reaction. Limited by imaging technology, we could not define the precise subcellular localization of the CRISP2‐acrosin complex in situ. However, the CRISP2‐acrosin signal was dispersed in the acrosome during capacitation. It is possible that the CRISP2‐acrosin complex is involved in the paracrystalline patches that are present in the acrosomal matrix.^16^ The consistent localization of CRISP2‐acrosin complexes in the sperm tail corresponded to the presence of ∼53 kDa proacrosin in the immunoblots of CRISP2 precipitates. In line with this, the ∼53 kDa proacrosin was the predominant form associated with sperm tail fractions. CRISP2 might function in retaining the ∼53 kDa proacrosin inactively in the sperm tail thereby providing an explanation for the stiffness of the midpiece in Crisp2^−/−^ sperm.^25^

Our findings that acrosomal proteins interact with CRISP2 may suggest that perinuclear theca proteins (the site of CRISP2) can interact with acrosomal proteins in spermatozoa. Consistent with this hypothesis, a robust study on another perinuclear theca protein, calicin, demonstrated an interaction with the inner acrosomal membrane and nuclear envelope proteins.^59^ Likewise, the identification that mitochondrial functional proteins can interact with CRISP2 may indicate that the ODF proteins are also interacting with mitochondrial proteins. The interaction of the ODF with the mitochondria in the midpiece has been recently reported for sperm from pigs and other mammalian species.^60^ Notably, in the list of the most abundant proteins identified in the CRISP2 eluate and native gel plugs, two molecular chaperones were also identified, which have been shown associated with human sperm during capacitation and may be involved in sperm‐ZP interaction.^61^

In addition to the proteomic identification of proteins that were present in the CRISP2‐positive protein complexes, it is important to realize that due to the condensed nature of protein structures in mature sperm cells, numerous additional minor proteins were detected. Both the excised 150 kDa band as well as CRISP2 co‐immuno precipitates may contain proteins that are not directly interacting with CRISP2 and are detected due to the high sensitivity of MS and low accuracy of gel band extraction. Despite the fact that two independent CRISP2 complex isolation techniques led to the identification of a similar CRISP2 interactome validation; all interacting proteins of interest require extensive biochemical validation as we have performed here for acrosin and ACRBP.

The observed redistributed CRISP2 interactions with both ACRBP and acrosin during the Ca^2+^ II acrosome reaction may well relate to the observed concomitant redistribution of CRISP2 and a Ca^2+^‐dependent dissociation of CRISP2‐protein interactions. It is well possible that other CRISP2 interacting proteins (as identified in this study with proteomics) also diminish their interactions with CRISP2 during the Ca^2+^ II acrosome reaction. A further dissociation of CRISP2 from the perinuclear theca post fertilization has been studied. It shows that within a few hours post fertilization (before any signs of decondensation of the sperm nucleus), CRISP2 from the perinuclear theca is dissociated and degraded in the oocyte's cytosol.^62^ The interactome with CRISP2 as identified in the current study can thus either during the Ca^2+^ II acrosome reaction or after fertilization become rapidly released from CRISP2‐containing protein complexes and thus become functional. However, the possible functions of those proteins, from the CRISP2‐containing protein complexes in the fertilization cascade should be experimentally explored in the future. Possible involvement of protein subunits from nucleosomes, proteasomes, splicosomes, RAB2B, and phospholipase C zeta enriched in the perinuclear theca are discussed in this context in a previous study.^53^

In summary, this study has identified ACRBP and acrosin as novel CRISP2 interacting partners in boar sperm. We have shown that the interaction between the target proteins was dynamic following capacitation suggesting that CRISP2 complexes play roles during capacitation. Our prior study implies that CRISP2 forms distinct protein complexes dependent on cysteine oxidation and oligomerization of the CAP domain regulated by Zn^2+^.^23^ CRISP2 uses both pathways scaffolding itself as well as other proteins contributing to the condensed structures of mammalian spermatozoa. In the future, the role of CRISP2 as a scaffolding protein in the formation and dissociation of protein complexes important for fertilization should be further explored. Altogether these discoveries enhance and expand the roles of CRISP2 as a protein that is involved in protein complex formation and dissociation, in sperm function, and in the fertilization process.

## AUTHOR CONTRIBUTIONS

Min Zhang performed the experiments, wrote the draft of the manuscript, and contributed to figure preparation and manuscript editing. Riccardo Zenezini Chiozzi conducted experiments, contributed to the analysis of the raw data, and reviewed the manuscript. Elizabeth G. Bromfield analyzed the data, contributed to figure preparation, and manuscript revision. AH contributed to the data collection, resource provision, and manuscript revision. J Bernd Helms contributed to supervision, provided resources, and reviewed the manuscript. Bart M Gadella conceived of the study, contributed to manuscript preparation, revision and editing, and supervision.

## CONFLICT OF INTEREST STATEMENT

The authors have no conflict of interests to disclose.

## Supporting information

Supporting Information

Supporting Information

## Data Availability

The mass spectrometry proteomics data in the study have been deposited to the ProteomeXchange Consortium via the PRIDE partner repository with the dataset identifier PXD037181.
