# Supplementary material for: Characterization of acrosin and acrosin binding protein as novel CRISP2 interacting proteins in boar spermatozoa
Source: Andrology. 2023 Mar 9;11(7):1460–71. doi: 10.1111/andr.13413 (PMC10947329; doi:10.1111/andr.13413)
Supplement: Supplementary file 1 — Supporting Information [file ANDR-11-1460-s002.docx]

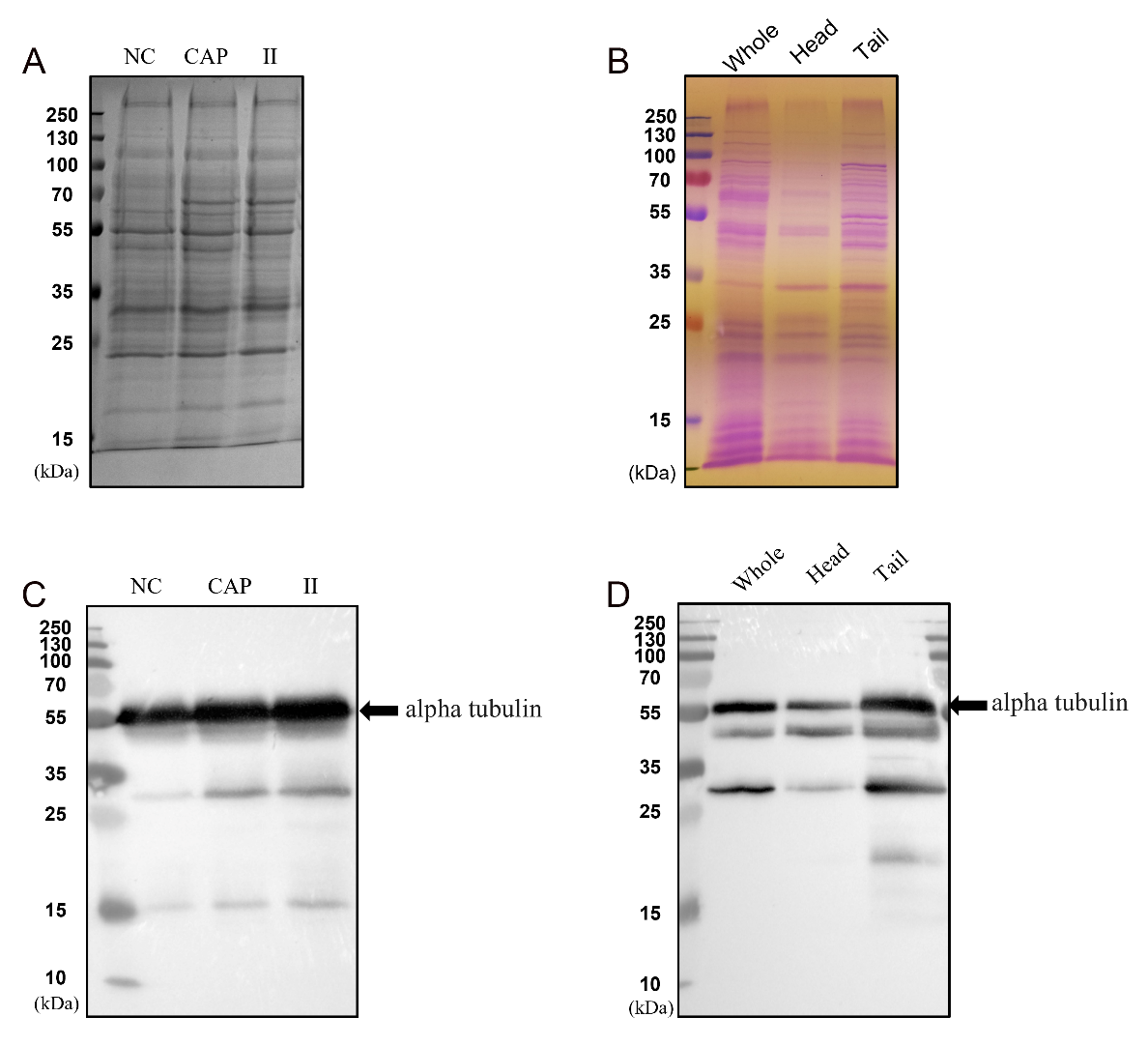


**Supplementary figure S1.** Same amount sperm cells were lysed in the same volume buffer and the same amount lysates were loaded on SDS-PAGE gels. (A) Lysates from NC, CAP and II sperm cells were analyzed by SDS-PAGE and coomassie blue staining. (B) Lysates from whole sperm cells/head/tail fractions were analyzed by SDS-PAGE and coomassie blue staining. (C) Western blots analysis of the lysates from NC, CAP and II sperm cells probed with alpha tubulin. (D) Western blots analysis of the lysates from whole sperm cells/head/tail fractions probed with alpha tubulin. Black arrows indicated the expected protein bands.

**
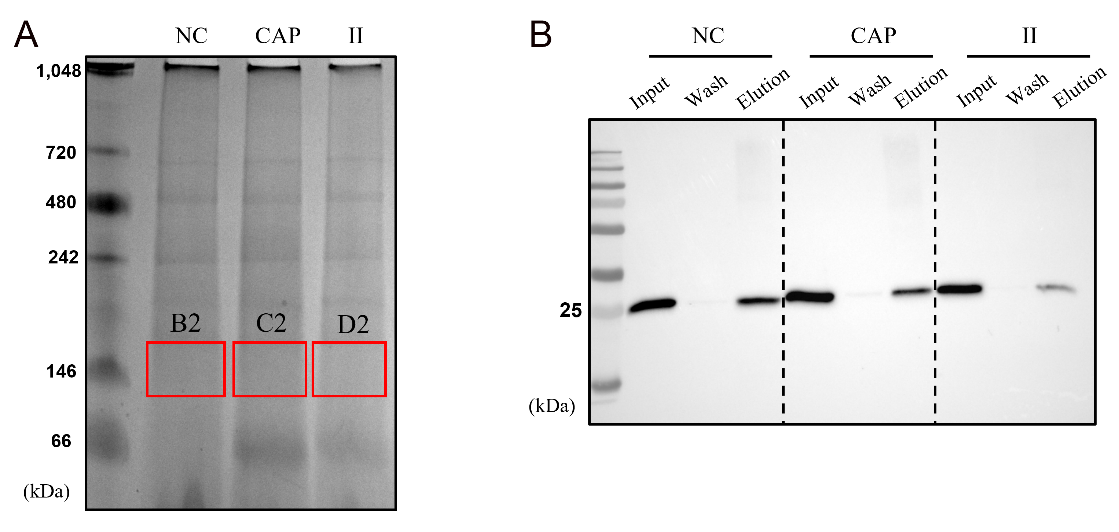
**

**Supplementary figure S2.** (A) Blue native PAGE analysis on the lysates from NC, CAP and II sperm cells. Gels in red tangle box (~150 kDa) were carefully sliced and prepared for MS analysis. (B) Validation of CRISP2 by western blots analysis on CRISP2 precipitates before MS analysis.

**
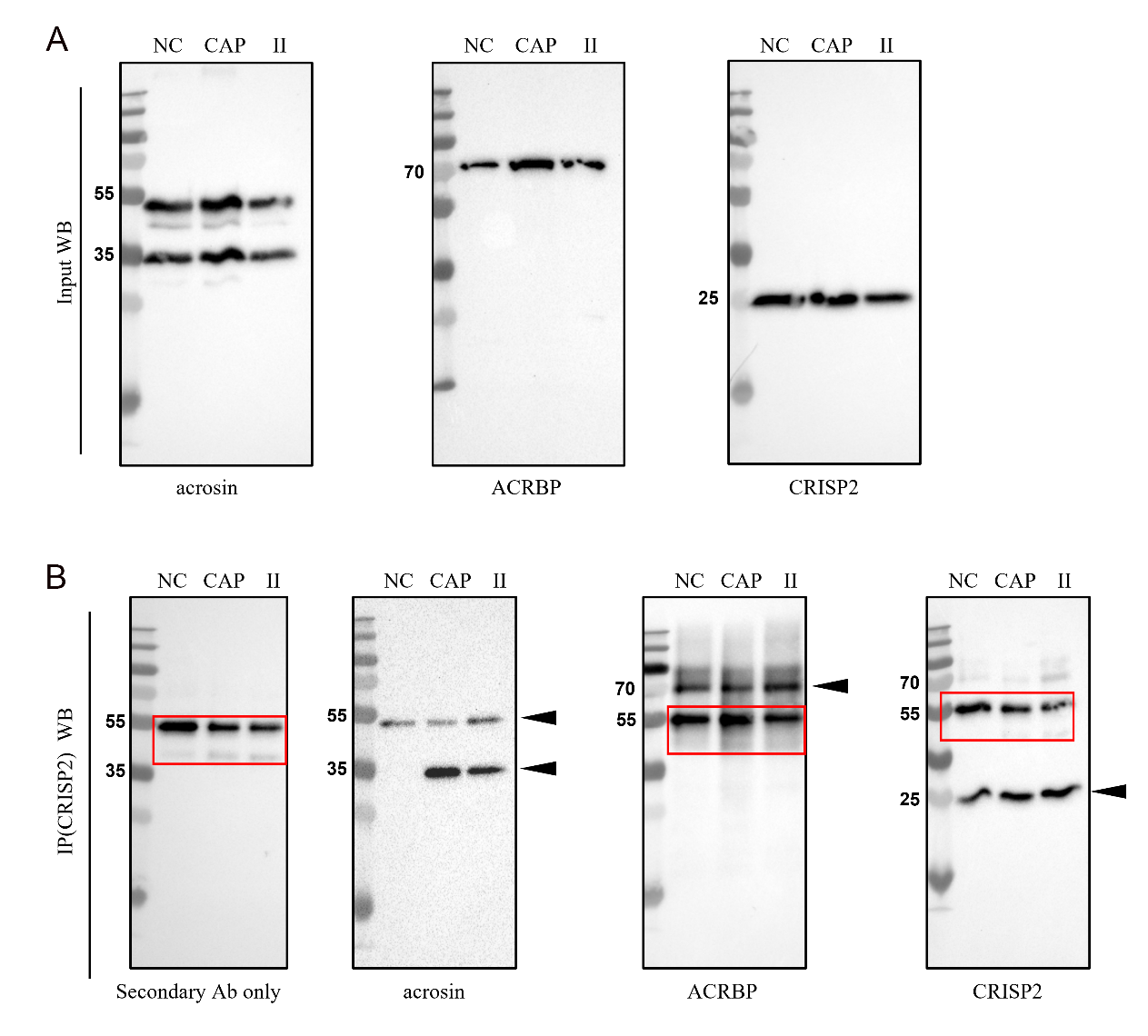
Supplementary figure S3.** Full western blots images corresponding to Figure 2. In Figure S3B, blots were incubated with secondary antibody only and bands in red tangle box indicated small contamination from primary antibody. Black arrows indicated the expected protein bands corresponding to input western blots.

**Supplementary Table S1. List of primary and secondary antibodies used in experiments.**

WB indicates western blot; IF indicates Immunofluorescence; IIF indicates Indirect Immunofluorescence.

| Primary antibodies | Supplier | Species | Type | Dilution  WB IIF | | Reference |
| --- | --- | --- | --- | --- | --- | --- |
| CRISP2 | Proteintech | rabbit | polyclonal | 1:1000 | 1:100 | 19066–1-A |
| acrosin (ACR-2) | Thermo Scientific | mouse | monoclonal | 1:1000 | 1:100 | MA1-19180 |
| ACRBP | antibodies-online | rabbit | polyclonal | 1:500 | 1:50 | ABIN5618703 |
| alpha tubulin | Abcam | rabbit | polyclonal | 1:500 |  | ab15246 |
| PNA Alexa Fluor 488 | Thermo Scientific | peanut |  | 5 μg/ml (IF) | | L21409 |

| **Secondary antibodies** | **Supplier** | **Dilution** |
| --- | --- | --- |
| Goat anti rabbit HRP | Jackson | 1:5000 |
| Goat anti mouse HRP | Jackson | 1:5000 |
| Goat anti rabbit Alexa Fluor 488 | Thermo Scientific | 1:100 |
| Goat anti rabbit Alexa Fluor 568 | Thermo Scientific | 1:100 |
| Goat anti mouse Alexa Fluor 568 | Thermo Scientific | 1:100 |
